# Feasibility of assessing ultra-short-term pulse rate variability from video recordings

**DOI:** 10.7717/peerj.8342

**Published:** 2020-01-07

**Authors:** Miha Finžgar, Primož Podržaj

**Affiliations:** Faculty of Mechanical Engineering, University of Ljubljana, Ljubljana, Slovenia

**Keywords:** Remote photoplethysmography, Pulse rate variability, Signal processing, Pulse rate, Image processing, Biomedical engineering

## Abstract

**Objectives:**

Remote photoplethysmography (rPPG) is a promising non-contact measurement technique for assessing numerous physiological parameters: pulse rate, pulse rate variability (PRV), respiratory rate, pulse wave velocity, blood saturation, blood pressure, etc. To justify its use in ultra-short-term (UST) PRV analysis, which is of great benefit for several healthcare applications, the agreement between rPPG- and PPG-derived UST-PRV metrics was studied.

**Approach:**

Three time-domain metrics—standard deviation of normal-to-normal (NN) intervals (SDNN), root mean square of successive NN interval differences (RMSSD), and the percentage of adjacent NN intervals that differ from each other by more than 50 ms (pNN50)—were extracted from 56 video recordings in a publicly available data set. The selected metrics were calculated on the basis of three groups of 10 s recordings and their average, two groups of 30 s recordings and their average, and a group of 60 s recordings taken from the full-length recordings and then compared with metrics derived from the corresponding reference (PPG) pulse waveform signals by using correlation and effect size parameters, and Bland–Altman plots.

**Main results:**

The results show there is stronger agreement as the recording length increases for SDNN and RMSSD, yet there is no significant change for pNN50. The agreement parameters reach *r* = 0.841 (*p* < 0.001), *r* = 0.529 (*p* < 0.001), and *r* = 0.657 (*p* < 0.001), estimated median bias −1.52, −2.28 ms and −1.95% and a small effect size for SDNN, RMSSD, and pNN50 derived from the 60 s recordings, respectively.

**Significance:**

Remote photoplethysmography-derived UST-PRV metrics manage to capture UST-PRV metrics derived from reference (PPG) recordings well. This feature is highly desirable in numerous applications for the assessment of one’s health and well-being. In future research, the validity of rPPG-derived UST-PRV metrics compared to the gold standard electrocardiography recordings is to be assessed.

## Introduction

Heart-rate variability (HRV) concerns the fluctuations of interbeat intervals (IBIs), that is, the time intervals between two consecutive successive heartbeats (Shaffer & Ginsberg, 2017). It largely depends on regulation by the autonomic nervous system (ANS) of the cardiovascular system (Rajendra Acharya et al., 2006). Therefore, the level of HRV gives information about the ANS’ functionality and the heart’s ability to respond to it (Rajendra Acharya et al., 2006). The HRV standard defines long-term (LT; 24 h) and short-term (ST; 5 min) HRV assessment by means of time-domain, frequency-domain, and non-linear metrics (Malik et al., 1996). The gold standard for HRV measurements is electrocardiography (ECG).

Ultra-short-term (UST; <5 minutes) HRV has been investigated as an alternative to standard HRV analysis (for a full review of this topic, see Pecchia et al. (2018)). One of the most relevant studies considering the validity of UST recordings for HRV measurements was conducted by Munoz et al. (2015). The authors reported that an average of multiple 10 s recordings efficiently captures the standard deviation of normal-to-normal (NN) intervals (SDNN), whereas an average of multiple 10 s recordings or one single 30 s recording is recommended for accurately measuring the root mean square of successive NN interval differences (RMSSD). The study’s large sample (3,387 subjects) and the rigorous method applied to assess the validity of UST-HRV mean the study gives strong support for the adoption of these HRV metrics (Pecchia et al., 2018). Recently, Castaldo et al. (2019) examined the validity of UST-HRV metrics derived from 0.5-, 1-, 2-, 3-, and 5-minute-long ECG recordings. The authors reported that of the studied standardized HRV metrics, SDNN, the high-frequency component (HF), and standard deviation of Poincaré plot along the line of identity (SD2) derived from 1-, 2-, and 3-minute-long ECG recordings are valid surrogates for their ST-HRV counterparts. Of these three metrics, HF was identified as a good feature (accuracy exceeding 88%) for detecting mental stress. Most other studies did not statistically evaluate (e.g. only a correlation test) the results with sufficient rigor. This issue was identified by Shaffer & Ginsberg (2017), Londhe & Atulkar (2018) and is thoroughly addressed by Pecchia et al. (2018). The adoption (and standardization) of UST-HRV would be highly beneficial because in certain clinical applications, for example, routine check-ups and check-ups of patients with atrial fibrillation (Camm et al., 2010), ECG recordings of less than 5 min are recorded. Further, there is growing increasing demand for user-friendly (i.e. fast and comfortable) ways of assessing one’s well-being (mood, stress, health status) using UST-HRV when combined with wearable technology and smartphone applications. These applications require the lowest power consumption and computational load possible, making UST-HRV even more attractive.

Alongside the benefits of adopting UST-HRV, there is also a need to simplify the recording of the signals used in HRV assessment. Therefore, techniques that offer continuous measurements of pulse waveform signals, such as photoplethysmography (PPG) and impedance plethysmography, were proposed as surrogates for ECG. However, these techniques evaluate pulse rate variability (PRV) instead of HRV since the IBIs are based on pulse cycles and not on cardiac cycles like with ECG recordings. Schäfer & Vagedes (2013) report in their thorough review of the accuracy of assessing HRV by means of PRV that PRV may be used as an efficient, accurate estimator of HRV for healthy subjects at rest. Yet the level of agreement between HRV and PRV is lower in the case of physical or mental stress mostly due to motion artefacts. In these conditions, the ST-HRV metrics seem to be affected more than the LT-HRV ones (Schäfer & Vagedes, 2013).

Of all surrogate techniques for estimating PRV, remote photoplethysmography (rPPG) (Takano & Ohta, 2007; Verkruysse, Svaasand & Nelson, 2008; Hülsbusch, 2008) seems the most attractive. rPPG is an optical method of measuring minute skin-blood-volume changes, which are related to the cardiac cycle. The two key advantages of rPPG are: (1) the absence of physical contact between an rPPG measurement device (i.e. a digital camera) and a subject; and (2) the digital cameras are readily accessible even outside of ambulatory settings as they are built into smartphones, laptops, smart watches, vehicles, etc. The use of rPPG recordings in UST-PRV assessment is therefore a promising solution for many healthcare applications.

Table 1 lists the results of studies examining the validity of rPPG-derived ST- and UST-PRV metrics (hereinafter (U)ST-PRV metrics). The results show a strong correlation between the rPPG- and PPG-derived (U)ST-PRV metrics in most studies. The correlation is, on average, strongest for SDNN. However, except for Moreno et al. (2015) study, none of the studies statistically evaluated their results in a rigorous enough manner (for detailed information, see Table 1). Table 2 shows the characteristics (relevant to rPPG research) of the studies listed in Table 1. Building on the results and characteristics of the studies covered in the two tables (Tables 1 and 2), the following findings emerged: (U)ST-PRV was assessed by relying on recordings equal to or longer than 50 s, each study used a different frequency band while applying bandpass filtering, some of which did not even cover the entire range of expected PRs in humans ((0.7, 4) Hz (Poh, McDuff & Picard, 2011), (0.05, 4) Hz (Sun et al., 2012), (0.75, 3) Hz (McDuff, Gontarek & Picard, 2014), (1, 3) Hz (Moreno et al., 2015), (0.3, 6) Hz (Blackford, Piasecki & Estepp, 2016), (0.75, 4) Hz (Huang & Dung, 2016), (0.6, 2) Hz (Melchor Rodriguez & Ramos-Castro, 2018), (0.6, 2.8) Hz (Yu et al., 2018), (0.7, 3.5) Hz (Benezeth et al., 2018), (0.667, 2.5) Hz (Purtov et al., 2016), (0.1, 8) Hz (Iozzia, Cerina & Mainardi, 2016)), none of the studies applied a sufficiently rigorous method to justify the (U)ST-PRV measurements using rPPG (as mentioned) and, finally, to the best of our knowledge, none of them publicly shared their data set.

**Table 1:** Results of the studies assessing (ultra)-short-term pulse rate variability from video recordings. Results of the studies by Sun et al. (2012) and Cerina, Iozzia & Mainardi (2017) are shown for the maximum frame rate only, that is, 200 and 60 fps, respectively. ICC, intraclass correlation coefficient; IR, infrared; LRC, linear regression coefficient; MAE, mean absolute error; *n*, normalized values; *r*, correlation coefficient; *r*^2^, coefficient of determination; RMSE, root mean square error; SD, standard deviation; SD1, Poincaré plot standard deviation perpendicular to the line of identity; SD2, Poincaré plot standard deviation along the line of identity; SSE, sum of squared errors; VS, visible spectrum.

Study	Parameter	SDNN (ms)	RMSSD (ms)	pNN50 (%)	VLF (ms^2^ or n.u.)	LF (ms^2^ or n.u.)	HF (ms^2^ or n.u.)	LF/HF (%)	SD1 (ms)	SD2 (ms)
Poh, McDuff & Picard (2011)	Mean error	–	–	–	–	7.53 (*n*)	7.53 (*n*)	0.57	–	–
	SD of error	–	–	–	–	10.17 (*n*)	10.17 (*n*)	0.98	–	–
UST-PRV	RMSE	–	–	–	–	12.3 (*n*)	12.3 (*n*)	1.1	–	–
	*r*	–	–	–	–	0.92 (*n*)	0.92 (*n*)	0.88	–	–
Sun et al. (2012) ST-PRV	*r*	0.874*	–	–	–	0.971* (*n*)	0.978* (*n*)	0.875*	–	–
McDuff, Gontarek & Picard (2014)	*r*	–	–	–	–	0.93** (*n*)	0.93** (*n*)	0.93**	–	–
UST-PRV	RMSE	–	–	–	–	–	–	0.145	–	–
Moreno et al. (2015)	*r* (supine and sitting)	0.9544	0.8398	0.8635	0.9975/0.8928 (*n*)	0.9849/0.7934 (*n*)	0.9844/0.7838 (*n*)	0.8662	0.8398	0.9734
		0.9108	0.518	0.538	0.9979/0.8928 (*n*)	0.9885/0.7934 (*n*)	0.99/0.7838 (*n*)	0.3186	0.518	0.9858
ST-PRV	Bias (supine and sitting)	4.12	11.5	8.66	21.73/−2.18 (*n*)	7.49/−0.53 (*n*)	73.26/2.72 (*n*)	−0.07	8.13	2.47
		6.68	19.3	16.87	4.96/−6.65 (*n*)	13.69/−2.56 (*n*)	206.83/9.21 (*n*)	−0.19	13.65	3.12
	Effect size (supine and sitting)	0.524	0.707	0.643	0.222/0.050 (*n*)	0.006/0.050 (*n*)	0.145/0.185 (*n*)	0.226	0.707	0.244
		0.675	0.699	0.684	0.105/0.522 (*n*)	0.280/0.285 (*n*)	0.627/0.468 (*n*)	0.257	0.669	0.631
Blackford, Piasecki & Estepp (2016) ST-PRV	*r*^2^	0.9212	–	–	–	0.9728	0.7789	–	–	–
	SSE	7.3	–	–	–	0.18	0.38	–	–	–
	Bias	12	–	–	–	0.13	0.78	–	–	–
Huang & Dung (2016) UST-PRV	MAE	0.49–3.79	0.09–7.97	–	–	–	–	–	0.03–5.73	0.38–5.34
		2.08–28.87	10.29–50.04	–	–	–	–	–	3.44–36.03	0.36–28.38
Iozzia, Cerina & Mainardi (2016) ST-PRV	*r* (rest and stand)	0.95	0.92	0.90	–	0.92 (*n*)	0.91 (*n*)	0.93	–	–
		0.82	0.75	0.68	–	0.63 (*n*)	0.76 (*n*)	0.71	–	–
Purtov et al. (2016) ST-PRV	*r*	–	–	–	*>*0.9	*>*0.9	–	–	–	–
Cerina, Iozzia & Mainardi (2017) ST-PRV	*r*	0.9532	0.9433	0.9124	–	0.8368 (*n*)	0.8372 (*n*)	–	–	–
Melchor Rodriguez & Ramos-Castro (2018) UST-PRV	*r* (stationary and motion)	0.9750*	0.9791*	0.9350*	–	0.9824* (*n*)	0.9824* (*n*)	0.9521* (*n*)	–	–
		0.9896*	0.9463*	0.9204*	–	0.9288* (*n*)	0.9302* (*n*)	0.8548* (*n*)	–	–
	ICC (stationary and motion)	0.9491*	0.9208*	0.8996*	–	0.9546* (*n*)	0.9546* (*n*)	0.7593* (*n*)	–	–
		0.9766*	0.8898*	0.8560*	–	0.9247* (*n*)	0.9265* (*n*)	0.4468*** (*n*)	–	–
	MAE (stationary and motion)	3.49	7.13	8.65	–	4.78 (*n*)	4.77 (*n*)	0.31 (*n*)	–	–
		2.65	7.81	7.80	–	5.54 (*n*)	5.47 (*n*)	1.10 (*n*)	–	–
	RMSE (stationary and motion)	4.26	8.29	10.35	–	6.20 (*n*)	6.20 (*n*)	0.61 (*n*)	–	–
		3.34	9.34	10.30	–	7.44 (*n*)	7.34 (*n*)	3.28 (*n*)	–	–
Yu et al. (2018) ST-PRV	LRC	0.99 (VS)	–	–	–	1.09 (VS)	1.12 (VS)	1.10 (VS)	–	–
		0.95 (IR)	–	–	–	1.11 (IR)	1.07 (IR)	1.09 (IR)	–	–
Benezeth et al. (2018) UST-PRV	Accuracy	–	–	–	–	98.25%	97.59%	96.90%	–	–

**Notes:**

* *p* < 0.001.

** *p* < 0.01.

*** *p* < 0.05.

**Table 2:** Overview of the characteristics of studies assessing (ultra)-short-term pulse rate variability from video recordings. BVP, blood volume pulse; CFL, compact fluorescent light; CMOS, complementary metal-oxidesemiconductor; *d*, distance between camera and measuring site; DC, direct current; DLSR, digital single-lens reflex camera; ECG, electrocardiography; F, female; HF, high-frequency band; IR, infrared; LED, light-emitting diode; LF, low-frequency band; M, male; *N*_s_, number of subjects; pNN50, percentage of normal-to-normal (NN) intervals that differ by more than 50 ms; PRV, pulse rate variability; RGB, red, green, and blue; RGBCO, red, green, blue, cyan, and orange; RMSSD, root mean square of successive NN interval differences; SD1, Poincaré plot standard deviation perpendicular to the line of identity; SD2, Poincaré plot standard deviation along the line of identity; SDNN, standard deviation of the NN intervals; *t*, duration of the recordings; TP, total power; VLF, very-low-frequency band.

Study characteristics	
Poh, McDuff & Picard (2011)	
PRV metrics: LF, HF, LF/HF	Camera: RGB (15 fps)
*N*_s_: 12 (4F + 8M)	Lighting: daylight only
*t* (s): 60	Recording setup: sitting still
Measuring site: face	Skin color: different
*d* (m): 0.5	Reference measurement: BVP sensor (256 Hz)
Sun et al. (2012)	
PRV metrics: SDNN, LF, HF, LF/HF	Camera: monochrome CMOS (200 fps)
*N*_s_: 10 (3F + 7M)	Lighting: 2× IR light only
*t* (s): 240	Recording setup: rest, cushion placed under the left palm
Measuring site: left palm	Skin color: not given
*d* (m): 0.4	Reference measurement: pulse oximeter
McDuff, Gontarek & Picard (2014)	
PRV metrics: LF, HF, LF/HF	Camera: RGBCO DLSR (30 fps)
*N*_s_: 10 (7F + 3M)	Lighting: daylight and indoor illumination
*t* (s): 120	Recording setup: (1) sitting still, (2) sitting, performing mental arithmetic test
Measuring site: face	Skin color: Asian, Caucasian, Hispanic
*d* (m): 3	Reference measurement: BVP sensor
Moreno et al. (2015)	
PRV metrics: SDNN, RMSSD, pNN50, VLF, LF, HF, LF/HF	Camera: RGB (30 fps)
*N*_s_: 20 (5F + 15M)	Lighting: dedicated light illuminating the face
*t* (s): 300	Recording setup: (1) sitting, (2) lying (supine) still with eyes closed
Measuring site: face	Skin color: Caucasian
*d* (m): 0.8	Reference measurement: chest strap (1 kHz)
Blackford, Piasecki & Estepp (2016)	
PRV metrics: SDNN, LF, HF	Camera: RGB (29.97 fps)
*N*_s_: 19	Lighting: daylight and fluorescent indoor illumination
*t* (s): 300	Recording setup: sitting still
Measuring site: face	Skin color: not given
*d* (m): 25	Reference measurement: ECG (16 kHz downsampled to 1,200 Hz)
Huang & Dung (2016)	
PRV metrics: SDNN, RMSSD, SDSD, SD1, SD2	Camera: RGB frontal smartphone camera (30 fps)
*N*_s_: 6 (2F + 4M)	Lighting: not given
*t* (s): 60	Recording setup: (1) static, (2) occasional motion, (3) frequent motion
Measuring site: face	Skin color: not given
*d* (m): not given	Reference measurement: chest strap (unclear sampling rate)
Iozzia, Cerina & Mainardi (2016)	
PRV metrics: SDNN, RMSSD, pNN50, VLF, LF, HF, LF/HF	Camera: RGB camera (60 fps)
*N*_s_: 58	Lighting: daylight and artificial light
*t* (s): 300	Recording setup: rest-to-stand maneuver (i.e. from lying on bed to standing still)
Measuring site: face	Skin color: Fitzpatrick scale II-IV
*d* (m): not given	Reference measurement: ECG (256 Hz)
Purtov et al. (2016)	
PRV metrics: HF, LF, VLF, TP	Camera: RGB camera (30 fps)
*N*_s_: 20	Lighting: 2× fluorescent lights only
*t* (s): 1,200	Recording setup: sitting still
Measuring site: face	Skin color: not given
*d* (m): 3	Reference measurement: ECG (250 Hz)
Cerina, Iozzia & Mainardi (2017)	
PRV metrics: RMSSD, pNN50, HF	Camera: RGB (60 fps)
*N*_s_: 57	Lighting: daylight and artificial light (CFL bars)
*t* (s): 300	Recording setup: supine position
Measuring site: face	Skin color: Fitzpatrick scale II-IV
*d* (m): 1	Reference measurement: ECG (256 Hz)
Melchor Rodriguez & Ramos-Castro (2018)	
PRV metrics: SDNN, RMSSD, pNN50, LF, HF, LF/HF	Camera: RGB (15 fps)
*N*_s_: 15 (3F + 12M)	Lighting: daylight only
*t* (s): 50	Recording setup: (1) sitting still, (2) sitting, performing lateral and forward/backward movements
Measuring site: face	Skin color: fair skin
*d* (m): 0.3	Reference measurement: finger pulse sensor (1 kHz)
Yu et al. (2018)	
PRV metrics: SDNN, LF, HF	Camera: 2× monochrome camera, 1× RGB camera (R channel only) (50 fps)
*N*_s_: 4	Lighting: (1) white ambient light, (2) white ambient light + 1× red (625 nm) light, (3) white ambient light + 1× IR (850 nm) LED brick light
*t* (s): 300	Recording setup: lying still in supine position
Measuring site: face	Skin color: not given
*d* (m): 1.8	Reference measurement: ECG (500 Hz), PPG (125 Hz)
Benezeth et al. (2018)	
PRV metrics: VLF, LF, HF, (VLF+LF)/HF	Camera: RGB camera (50 fps)
*N*_s_: 16	Lighting: 2× direct DC lights
*t* (s): 120	Recording setup: sitting still, watching videos with (1) neutral, (2) emotional content
Measuring site: face	Skin color: not given
*d* (m): 1	Reference measurement: pulse oximeter

In this study, we wanted to investigate the extent to which the UST-PRV metrics derived from 10 s, 30 s, and 60 s rPPG recordings agree with the UST-PRV metrics derived from reference (PPG) recordings. The level of agreement was subjected to rigorous statistical evaluation. The studied PRV metrics were the time-domain metrics SDNN, RMSSD, and the percentage of adjacent NN intervals that differ from each other by more than 50 ms (pNN50). These metrics were extracted from facial video recordings and the corresponding reference (PPG) pulse waveform signals from the publicly available data set ‘Pulse rate detection data set’ (Stricker, Müller & Gross, 2014). In the ‘Materials and Methods’ section below, the data set used is presented, along with the procedure for extracting raw RGB signals from video recordings, the processing of these signals, detecting systolic peaks from the processed rPPG signals and extracting PRV metrics, while the steps in processing the reference signals and the approach to the statistical evaluation of the results are also explained.

## Materials and Methods

### Data set description

The publicly available ‘Pulse rate detection data set’ (PURE) (Stricker, Müller & Gross, 2014) was used in this study. It consists of 59 approximately 1-min-long facial video recordings (lossless compressed) of 10 subjects (two females and eight males) in six different controlled setups (data for subject no. 6 during setup two are missing in the data set). These setups include: (1) sitting still; (2) talking while avoiding head motion; (3) moving head parallel to the camera plane by following the moving rectangle on the screen; (4) same setup as the previous one, but with twice the speed of the moving rectangle; (5) head rotated by approximately 20° by looking at the targets in a predefined sequence; and (6) same setup as before, but with the rotation angle at around 35°. The videos were recorded using an eco274CVGE camera (SVS-Vistek GmbH’ Seefeld, Germany) at 30 fps a the resolution of 640 × 480. Reference pulse waveform signals were recorded using a finger clip pulse oximeter Pulox CMS50E (Novidion GmbH; Köln, Germany) at a sampling rate of 60 Hz. No special lighting was used. The average distance between the camera and each subject was 1.1 m. In the present study, a total of 56 sets containing video recordings and corresponding pulse waveform reference signals from the PURE data set was used. Three sets, that is, recordings of subject no. 5 in setups two and six, and of subject no. 7 in setup two, were eliminated due to noisy reference signals.

### rPPG signal extraction from the video recordings

The cascade object detector using the Viola-Jones frontal face detection algorithm (Viola & Jones, 2001) was employed to detect the face in the first video frame of each recording. The width of the bounding box containing the face was reduced to 60% of the original width (Poh, McDuff & Picard, 2011) to lower the number of non-skin pixels inside the bounding box. To track the facial region inside the modified bounding box, the Kanade-Lucas-Tomasi (Lucas & Kanade, 1981; Tomasi & Kanade, 1991) algorithm was applied. This algorithm first identifies the feature points, that is, good features to track (Shi & Tomasi, 1994), to provide reliable tracking. A point tracker then locates each point from the previous frame (i.e. old points) in the current one (i.e. new points). Upon finding all the points, the affine transformation parameters between the old and new points are estimated. The forward-backward error threshold (Kalal, Mikolajczyk & Matas, 2010) was set to two pixels to eliminate those points that could not be reliably tracked and thus to ensure the tracker's more robust performance. The obtained affine transformation parameters were then applied to the bounding box containing the face. Raw RGB signals were obtained from the spatial averaging of the RGB pixel values inside the bounding box in each frame (Verkruysse, Svaasand & Nelson, 2008; De Haan & Jeanne, 2013). Next, the raw RGB signals were resampled using a spline interpolation according to the given time-stamps of each frame as suggested in Wang, Stuijk & De Haan (2017), windowed using window length *l* = 256, and temporally normalized (De Haan & Jeanne, 2013). The extraction of the rPPG signals from the temporally normalized RGB signals was done using the recently proposed Continuous-Wavelet-Transform-based Sub-Band rPPG (SB-CWT) (Finžgar & Podržaj, 2018). This method algorithmically increases the dimensionality of RGB signals by means of a continuous-wavelet-transform-based decomposition using generalized Morse wavelets. We set the following values of the required input parameters in the SB-CWT: symmetry parameter γ = 3, time-bandwidth product *p*^2^ = 115, and number of voices per octave ν = 10. For the reasoning behind the choice of the selected values, we refer to Finžgar & Podržaj (2018). SB-CWT outperformed the state-of-the-art Sub-Band rPPG (SB) method (Wang et al., 2017b) in terms of signal-to-noise ratio (SNR) and the agreement between the average pulse rates (PRs) estimated from rPPG and the reference PRs (Finžgar & Podržaj, 2018). On the other hand, SB outperformed its direct algorithmic alternative, the plane-orthogonal-to-skin (POS) algorithm (Wang et al., 2017b) in terms of SNR in almost all categories (skin-tone, light source, luminance level and miscellaneous motion scenarios) for all window lengths (*l* = 32, *l* = 64, *l* = 128 and *l* = 256), whereas POS showed the best overall performance in terms of SNR when compared with seven state-of-the-art rPPG algorithms (Wang et al., 2017a): G (Verkruysse, Svaasand & Nelson, 2008), G-R (Hülsbusch, 2008), PCA (Lewandowska et al., 2011), ICA (Poh, McDuff & Picard, 2010), CHROM (De Haan & Jeanne, 2013), PBV (De Haan & Van Leest, 2014), and 2SR (Wang, Stuijk & De Haan, 2016). Therefore, we decided to use SB-CWT to extract the pulse waveform signals from the video recordings.

### Processing of the rPPG signals and extraction of the systolic peaks

**Figure 1:**
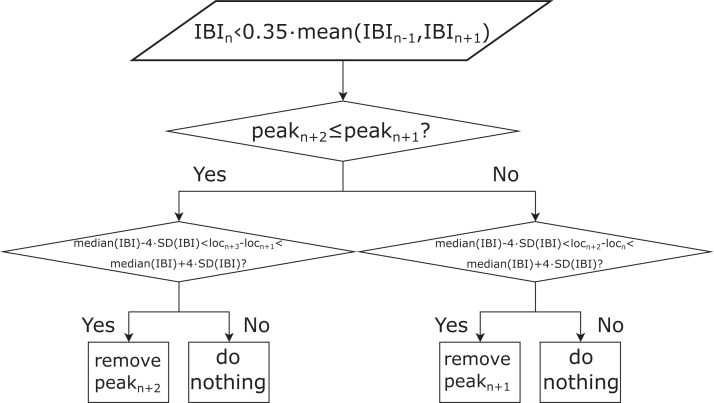
Schematic representation of the procedure for interbeat interval (IBI) filtering. loc_*n*_: timestamp of the *n*-th peak (*n* = 2: *N−*2, where *N* is the total number of IBIs), mean (IBI_*n−*1_, IBI_*n*+1_): mean length of the *n −* 1-th and *n* + 1-th IBI; median (IBI): median length of all the IBIs, peak_*n*_: height of the *n*-th detected peak in the pulse waveform signal, IBI_*n*_: the *n*-th IBI, SD(IBI): standard deviation of all IBIs.

The rPPG signals were then interpolated to 60 Hz in order to match the sampling rate of the reference PPG signals. In the next step, the systolic peaks of the resampled rPPG signals were detected using the derivative-based method for finding local maxima (MATLAB^®^ function findpeaks) with the minimum peak-to-peak distance set to 0.25 s, which corresponds to the upper frequency of the selected human pulse rate (PR) band ((40, 240) BPM). In so doing, we did not adapt our peak detection algorithm to the data set, but kept it in a form that makes it applicable to any data set, regardless of the HRs/PRs of the subjects. The IBI series was calculated from the detected peaks. In the next step, all IBIs shorter than 35% (experimentally set value) of the mean value of the preceding and following IBIs were identified. Based on these IBIs the peaks corresponding to the ectopic beats or noise were eliminated. The process of eliminating these peaks is shown in Fig. 1. All pulse waveform signals and corresponding refined peaks were visually inspected in order to evaluate the IBI filtering method’s performance. After the filtering, the final IBI series was obtained.

### Processing of the reference PPG signals

The reference PPG signals were first bandpass-filtered using a zero-phase first-order Butterworth filter with a lower and upper cut-off frequency of 0.67 and 3 Hz, respectively (these frequencies correspond to 40 and 180 BPM, which cover all the PRs present in the data set) and then resampled to 60 Hz. As the video recordings, the recorded reference signals were namely sampled non-uniformly and thus resampled according to the corresponding timestamps of each recording. The process of systolic peak detection and refinement of the IBIs for reference pulse waveform signals was the same as for the rPPG signals (see Fig. 1), with the only exception that the minimum peak-to-peak distance was set to 0.33 s, which corresponds to the upper cut-off frequency of the applied bandpass filter. To increase the SNR of the reference signals, we decided to apply the upper cut-off frequency of the applied bandpass filter that corresponds to the PRs present in the data set used. This was done in order to improve the SNR of the reference (PPG) pulse waveform signals. Similarly, the minimum peak-to-peak distance in the algorithm for finding peaks in the PPG signal was selected. In contrast to the processing of the pulse waveform signals extracted from the video recordings, the processing of reference pulse waveform signals was not adapted to the limits of the human PR band but to the limits of the data set being used.

### Extraction of PRV metrics from rPPG and reference PPG signals

Three time-domain metrics—SDNN, RMSSD, and pNN50—were studied. SDNN relates to all the cyclic components that affect the variability over the entire duration of the recording (Malik et al., 1996), RMSSD is associated with the short-term components of HRV, that is, to high-frequency variations (Malik et al., 1996), while pNN50 is correlated with peripheral nervous system activity and RMSSD (Shaffer & Ginsberg, 2017). SDNN and RMSSD were calculated for six different recording lengths (three non-overlapping 10 s groups, two 30 s groups, and one group of 60 s recordings) extracted from each of the 56 rPPG recordings. The first 10 s, the first 30 s, and the 60 s groups of recordings started at the third detected peak in the full recording, the second 10 s and the second 30 s groups of recordings started at the peak closest to the middle of the full recording, whereas the third 10 s group of recordings started at the peak closest to 4/5 of the length of the full recording. In addition, the average SDNN and RMSSD of the three 10 s groups of recordings (avg10s) and the two 30 s recordings’ groups (avg30s) were calculated. pNN50 was evaluated on the two 30 s, and 60 s groups of recordings. Further, the average pNN50 of the two 30 s recordings was calculated.

### Statistical analysis of the results

To test the effect of the IBI filtering refinement algorithm depicted in Fig. 1 on the accuracy of the SDNN, RMSSD, and pNN50 estimation, the relative change in the mean absolute error (MAE) before and after filtering was calculated.

The level of agreement of the rPPG-derived PRV metrics with the reference-derived ones was assessed for the raw (all three studied metrics) as well as for the log-transformed data (SDNN and RMSSD only). For the non-normally distributed data, Spearman’s coefficient, the Bland–Altman (BA) plot for non-normally distributed data (estimated bias defined as the median of the differences instead of the mean) and Cliff’s delta were used and, in the case of normally distributed data, Pearson’s correlation coefficient, the BA plot for normally distributed data and Cohen’s *d*. The described procedure is based on the recommendations given by of Pecchia et al. (2018). To test the normality of the data, the Shapiro–Wilk test was used.

## Results

We first tested if any differences existed between the six different recording setups in terms of mean SDNN, RMSSD, and pNN50 derived from the 60 s reference (PPG) recordings. Results of ANOVA showed statistically significant differences between the group means (SDNN: *p* = 0.4629, RMMSD: *p* = 0.9307, pNN50: *p* = 0.7996). This finding justifies the decision to take all the recordings and then form a single group of recordings. The results also indicate that none of the setups elicited a change in the physiological state of the subject that would be reflected in a significant change in SDNN, RMSSD, or pNN50. Next, we performed a multiple comparison of the group means using Bonferroni critical values, comparing the SDNN, RMSSD, and pNN50 values derived from the 60 s rPPG recordings with the corresponding values derived from the reference (PPG) recordings. The difference between the estimated group means is the smallest for setup one (sitting still) and the biggest for setup two (talking, while avoiding head motion) for all studied UST-PRV metrics.

Table 3 shows the relative change in MAE of SDNN, RMSSD, and pNN50 before and after filtering the IBIs. The results indicate that after filtering the IBIs the MAE diminished for all recording lengths and PRV metrics. The drop in MAE is on average greater for the longer recording lengths because such recordings are more likely to contain more artefacts.

**Table 3:** Comparison of the relative changes in mean absolute error (MAE) of SDNN, RMSSD, and pNN50 before and after the IBI filtering.

	10s-1	10s-2	10s-3	30s-1	30s-2	60s
SDNN	−0.423	−0.356	−0.374	−0.639	−0.473	−0.719
RMSSD	−0.365	−0.355	−0.486	−0.511	−0.473	−0.537
pNN50	–	–	–	−0.061	−0.054	−0.053

**Note:**

10s-1, 10s-2, and 10s-3 denote the first, second, and third groups of 10 s recordings, 30s-1 and 30s-2 the first and second groups of 30 s recordings, and 60s the group of 60 s recordings.

The results of the Shapiro–Wilk test for all raw rPPG-derived PRV metrics rejected the null-hypothesis that the population is normally distributed at α = 0.05. In the case of the log-transformed SDNN (lnSDNN) data, the null-hypothesis was not rejected, whereas it was rejected for the log-transformed RMSSD data (lnRMSSD), except for the third group of 10 s recordings. Log-transformation was not applied to the pNN50. In further analysis, SDNN, lnSDNN, RMSSD, and pNN50 were included. The parametric statistical models were used for the analysis of lnSDNN, whereas the non-parametric ones were used for SDNN, RMSSD, and pNN50.

Table 4 shows the SDNN, lnSDNN, RMSSD, and pNN50 values (expressed as a mean *±* standard deviation) derived from the rPPG and corresponding reference recordings of different lengths and the MAEs. Results of a one-way ANOVA show no statistically significant differences (α = 0.05) between the group means of SDNN, lnSDNN, RMSSD, and pNN50 between PPG and rPPG for any of the studied recording lengths. MAE tends to decrease with an increasing recording length for all studied UST-PRV metrics. The MAE of RMSSD exceeds that of SDNN.

**Table 4:** Values of SDNN, lnSDNN, RMSSD, and pNN50 (expressed as a mean ± standard deviation) derived from the rPPG and reference (PPG) recordings together with the corresponding mean absolute errors (MAEs). 10s-1, 10s-2, and 10s-3 denote the first, second, and third groups of 10 s recordings, 30s-1 and 30s-2 the first and second groups of 30 s recordings, 60s the group of 60 s recordings, avg10s the average of the three groups of 10 s recordings, and avg30s the average of the two groups of 30 s recordings, ref: reference.

	10s-1	10s-2	10s-3	avg10s	30s-1	30s-2	avg30s	60s
SDNN_ref_ (ms)	45.46 ± 21.67	39.24 ± 19.70	42.08 ± 25.28	42.26 ± 16.85	46.97 ± 17.03	44.40 ± 19.66	45.68 ± 16.24	48.61 ± 16.99
SDNN_rPPG_ (ms)	44.79 ± 24.91	38.71 ± 20.89	37.85 ± 24.83	40.45 ± 17.67	48.02 ± 20.38	41.49 ± 19.40	44.76 ± 17.85	48.49 ± 19.81
MAE_SDNN_ (ms)	11.68	9.60	10.18	8.53	8.87	8.78	8.43	8.14
lnSDNN_ref_	3.69 ± 0.53	3.55 ± 0.50	3.59 ± 0.54	3.66 ± 0.41	3.78 ± 0.40	3.70 ± 0.44	3.76 ± 0.38	3.82 ± 0.38
lnSDNN_rPPG_	3.68 ± 0.50	3.53 ± 0.51	3.49 ± 0.53	3.62 ± 0.41	3.78 ± 0.43	3.63 ± 0.44	3.72 ± 0.40	3.80 ± 0.41
MAE_lnSDNN_	0.27	0.26	0.27	0.20	0.19	0.19	0.18	0.16
RMSSD_ref_ (ms)	44.45 ± 28.21	42.33 ± 23.70	43.28 ± 25.33	43.36 ± 21.91	43.35 ± 21.96	47.70 ± 28.67	45.53 ± 23.10	46.27 ± 23.78
RMSSD_rPPG_ (ms)	45.02 ± 35.77	41.31 ± 25.96	39.92 ± 20.23	42.08 ± 22.44	46.02 ± 28.15	42.84 ± 23.41	44.43 ± 24.08	46.40 ± 27.41
MAE_RMSSD_ (ms)	21.56	15.80	15.15	14.19	17.48	16.74	16.35	16.77
pNN50_ref_ (%)	–	–	–	–	24.02 ± 24.02	24.01 ± 24.01	24.02 ± 24.02	24.14 ± 24.14
pNN50_rPPG_ (%)	–	–	–	–	20.89 ± 20.89	20.46 ± 20.46	20.68 ± 20.68	20.77 ± 20.77
MAE_pNN50_ (%)	–	–	–	–	11.46	10.70	10.24	10.15

Table 5 shows the values of the correlation coefficients and effect sizes for SDNN, lnSDNN, RMSSD, and pNN50 derived from rPPG recordings of different lengths and the corresponding reference recordings. The highest correlation is achieved for SDNN, the lowest for RMSSD. Values of Spearman coefficients (*r*_*s*_) and Pearson correlation coefficients (*r*) tend to rise with an increasing recording length for all metrics studied. All correlation parameters are statistically significant at α = 0.05. Cliff’s delta (*d*_cliff_) and Cohen’s d (*d*_cohen_) tend to decrease as the recording length increases for SDNN, RMSSD, and lnSDNN.

**Table 5:** Correlation and effect size metrics measuring agreement between the SDNN, lnSDNN, RMSSD, and pNN50 values derived from the rPPG recordings and corresponding reference (PPG) recordings.

	10s-1	10s-2	10s-3	avg10s	30s-1	30s-2	avg30s	60s
Spearman coefficient*								
SDNN	0.708	0.803	0.750	0.763	0.766	0.778	0.761	0.841
RMSSD	0.358	0.536	0.507	0.536	0.484	0.579	0.544	0.529
pNN50	–	–	–	–	0.584	0.679	0.666	0.657
Pearson correlation coefficient*								
lnSDNN	0.654	0.787	0.790	0.776	0.737	0.787	0.790	0.842
Cliff’s delta								
SDNN	0.060	0.041	0.124	0.106	0.015	0.100	0.062	0.050
RMSSD	−0.003	0.098	0.015	0.047	−0.011	0.104	0.061	0.068
pNN50	–	–	–	–	0.082	0.080	0.084	0.094
Cohen’s *d*								
lnSDNN	0.029	0.050	0.200	0.114	−0.013	0.160	0.080	0.045

**Note:**

* *p* < 0.05; 10s-1, 10s-2, and 10s-3 denote the first, second, and third group, of 10 s recordings, 30s-1 and 30s-2 the first and second group, of 30 s recordings, 60s the group of 60 s recordings, avg10s the average of the three groups of 10 s recordings, and avg30s the average of the two groups of 30 s recordings.

**Figure 2:**
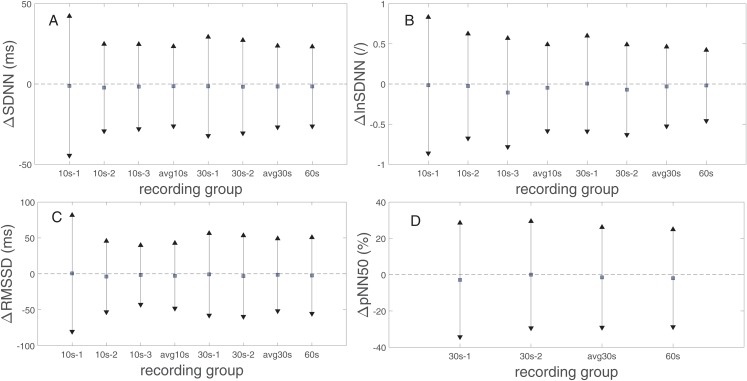
Biases and 95% limits of agreement of (A) SDNN, (B) lnSDNN, (C) RMSSD, and (D) pNN50 values derived from rPPG recordings of different lengths compared to the reference (PPG) recording equivalents. The squares represent estimated biases (expressed as median in (A), (C), (D) and as mean values in (B)). The solid lines between the upward- and downward-pointing triangles represent the intervals defined by the 95% limits of agreement (LoA).

Figure 2 presents the biases and 95% limits of the agreement (LoA) of SDNN, lnSDNN, RMSSD, and pNN50 values derived from the rPPG recordings of different lengths compared to the reference (PPG) recording equivalents. The estimated bias is negative for all PRV metrics and all recording lengths, except for the 30s-1 lnSDNN and 10s-1 RMSSD. The biases are smaller for SDNN than for RMSSD. The LoA tend to decrease as the recording length increases for all studied UST-PRV metrics.

**Figure 3:**
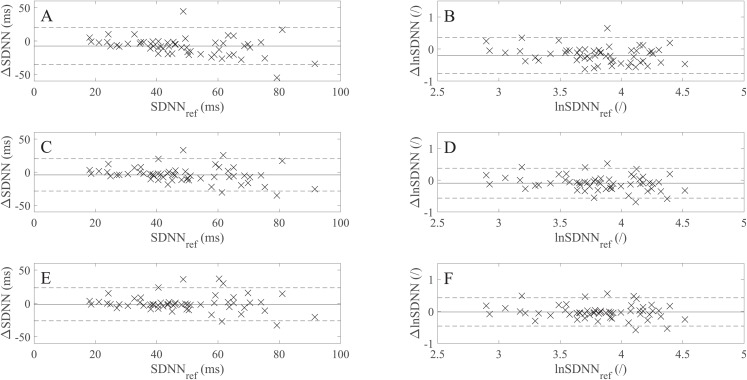
Bland–Altman (BA) plots showing levels of agreement between the SDNN/lnSDNN values derived from rPPG and those derived from the reference (PPG) signals. (A)–(F) show the BA plots for (A) SDNN derived from the average of all 10 s recordings, (B) lnSDNN derived from the average of all 10 s recordings, (C) SDNN derived from the average of all 30 s recordings, (D) lnSDNN derived from the average of all 30 s recordings, (E) SDNN derived from the 60 s recordings and (F) lnSDNN derived from the 60 s recordings. ∆lnSDNN: the difference between the log-transformed rPPG- and reference-derived SDNN, ∆SDNN: the difference between the raw rPPG- and reference-derived SDNN, lnSDNN_ref_: log-transformed reference-derived SDNN, SDNN_ref_: raw reference-derived SDNN. Dashed lines in (A)–(F) denote the upper and lower 95% limits of agreement, solid lines in (A), (C) and (E) denote the estimated bias expressed as a median value while the solid lines in (B), (D) and (F) denote the estimated bias, expressed as a mean value.

Table 6 shows the SDNN, lnSDNN, RMSSD, and pNN50 values (expressed as a mean *±* standard deviation) derived from rPPG recordings of different lengths and the 60 s PPG (reference) recordings and the corresponding MAEs. As the recording length increases, the mean of all studied metrics increases. The increase in the mean value is more prominent for SDNN (8.04 ms from avg10s to the 60 s recordings) than for RMSSD (4.32 ms from avg10s to 60 s recordings), whereas for pNN50 the increase is negligible. When it comes to the relation between MAE and recording length, the MAE of SDNN, lnSDNN, and RMSSD decreases, whereas such a trend is not observable for pNN50. Figure 3 presents the BA plots showing the agreement between SDNN/lnSDNN derived from the avg10s, avg30s, and 60 s rPPG recordings and SDNN/lnSDNN derived from the 60 s PPG recordings. The estimated bias is negative for all recording lengths, and decreases with an increasing recording length for both SDNN (from −7.51 ms for avg10s to −1.52 ms for the 60 s recordings) and lnSDNN (from −0.201 for avg10s to −0.018 for the 60 s recordings). The width of the 95% limits of agreement also decreases as the recording length increases for SDNN (from −35.215–20.184 ms for avg10s to −26.274–23.236 ms for the 60 s recordings) and lnSDNN (from −0.768–0.366 for avg10s to −0.458–0.422 for the 60 s recordings).

**Table 6:** Values of SDNN, lnSDNN, RMSSD, and pNN50 (expressed as a mean ± standard deviation) derived from rPPG recordings of different lengths and from the 60 s reference (PPG) recordings together with the corresponding mean absolute errors (MAEs). 10s-1, 10s-2, and 10s-3 denote the first, second and third groups of 10 s recordings, 30s-1 and 30s-2 the first and second groups of 30 s recordings, 60s the group of 60 s recordings, avg10s the average of all 10 s recordings, and avg30s the average of all 30 s recordings.

	SDNN (ms)	MAE_SDNN_ (ms)	lnSDNN	MAE_lnSDNN_	RMSSD (ms)	MAE_RMSSD_ (ms)	pNN50 (%)	MAE_pNN50_ (%)
PPG (reference)								
60s	48.61 ± 16.99	–	3.82 ± 0.38	–	46.27 ± 23.78	–	24.14 ± 19.11	–
rPPG								
10s-1	44.79 ± 24.91	16.73	3.68 ± 0.50	0.36	45.02 ± 35.77	23.00	–	–
10s-2	38.71 ± 20.89	16.79	3.53 ± 0.51	0.41	41.31 ± 25.96	16.29	–	–
10s-3	37.85 ± 24.83	17.24	3.49 ± 0.53	0.43	39.92 ± 20.23	14.47	–	–
avg10s	40.45 ± 17.67	11.96	3.62 ± 0.4	0.27	42.08 ± 22.44	14.77	–	–
30s-1	48.02 ± 20.38	11.12	3.78 ± 0.43	0.23	46.02 ± 28.15	18.31	20.89 ± 16.32	11.16
30s-2	41.49 ± 19.40	12.36	3.63 ± 0.44	0.28	42.84 ± 23.41	14.95	20.46 ± 18.22	10.76
avg30s	44.76 ± 17.85	9.55	3.72 ± 0.40	0.20	44.43 ± 24.08	16.10	20.68 ± 16.39	9.99

A comparison of the BA plots for the raw (Figs. 3B, 3D and 3F) and log-transformed data (Figs. 3A, 3C and 3E) show that the number of points lying outside LoA is the same for the avg10s (two points) and 60 s recordings (six points), whereas there is one more point lying outside LoA for avg30s in the case of the log-transformed data (five vs four points).

**Figure 4:**
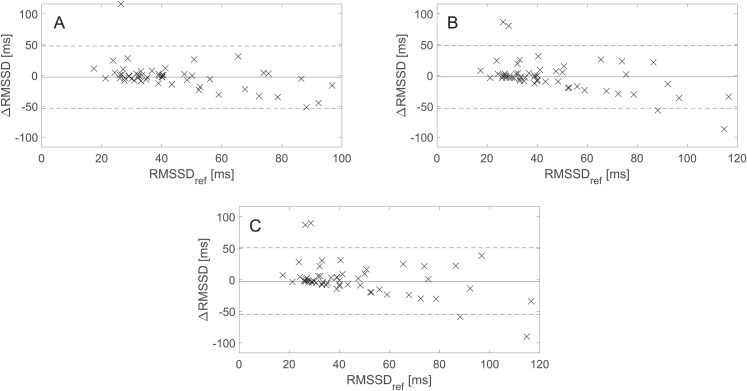
Bland–Altman (BA) plots showing agreements between the RMSSD values derived from rPPG and those derived from the reference (PPG) signals. (A)–(C) show the BA plots for RMSSD derived from (A) the average of all 10 s recordings, (B) the average of all 30 s recordings, and (C) the 60 s recordings. ∆RMSSD: the difference between the raw rPPG- and reference-derived RMSSD, RMSSD_ref_: raw reference-derived RMSSD. Dashed lines denote the upper and lower 95% limits of agreement, and solid lines indicate the estimated bias expressed as a median value.

Figure 4 presents the BA plots showing levels of agreement between the RMSSD values derived from the avg10s, avg30s, and 60 s rPPG recordings and the RMSSD values calculated from the 60 s rPPG recordings. Like with SDNN and lnSDNN, the estimated bias is negative for all recording lengths and tends to decrease as the recording length increases for RMSSD (from −2.59 ms for avg10s to −2.28 ms for the 60 s recordings). The trend of a decreasing mean with an increasing recording length is more prominent for SDNN than for RMSSD. In contrast to the SDNN/lnSDNN, the width of LoA grows with the an increasing recording length for RMSSD (from −53.00–47.82 ms for avg10s to −55.28–50.72 ms for the 60 s recordings).

**Figure 5:**
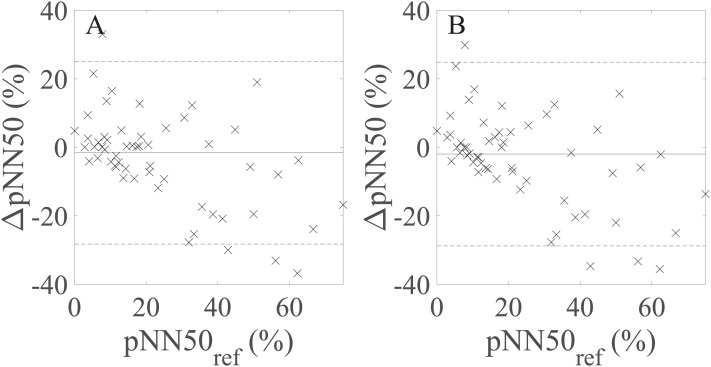
Bland–Altman plots showing agreement between the rPPG- and reference-derived pNN50 from (A) the average of all 30 s recordings and (B) the 60 s recordings. ∆pNN50: the difference between the raw rPPG- and reference-derived pNN50, pNN50_ref_: raw reference-derived pNN50. Dashed lines in denote the upper and lower 95% limits of agreement, whereas solid lines indicate the estimated bias expressed as a median value.

BA plots showing the agreement between pNN50 values derived from the avg30s/60 s rPPG recordings and pNN50 values calculated from the 60 s reference (PPG) recordings are shown in Fig. 5. There is no statistically significant difference at α = 0.05 between the estimated bias and the width of the LoA for the avg30s and 60 s recordings.

Table 7 shows the values of the correlation coefficients and effect size metrics. The values of *r*_*s*_ and *r* increase whereas *d*_cliff_ and *d*_cohen_ decrease as the recording length increases for SDNN, RMSSD, and lnSDNN. Values of *d*_cliff_ are close to zero for the avg30s and 60 s recordings for SDNN, RMSSD, and pNN50, meaning that the effect size is small. The effect size defined by *d*_cohen_ is small for the avg30s and 60 s recordings and moderate for avg10s. There is no significant change in the values of *r*_*s*_ and *d*_cliff_ as the recording length increases for pNN50. The highest correlation is achieved for lnSDNN, the lowest for RMSSD. All correlations are statistically significant.

**Table 7:** Correlation and effect size metrics measuring the agreement between the SDNN, lnSDNN, RMSSD, and pNN50 values derived from rPPG recordings of different lengths and the metrics derived from the 60 s reference (PPG) recordings.

	10s-1	10s-2	10s-3	avg10s	30s-1	30s-2	avg30s
Spearman coefficient*							
SDNN	0.555 (*p* < 0.001)	0.543 (*p* < 0.001)	0.599 (*p* < 0.001)	0.727 (*p* < 0.001)	0.733 (*p* < 0.001)	0.695 (*p* = 0.001)	0.800 (*p* < 0.001)
RMSSD	0.327 (*p* < 0.05)	0.496 (*p* < 0.001)	0.591 (*p* < 0.001)	0.536 (*p* < 0.001)	0.469 (*p* < 0.001)	0.591 (*p* < 0.001)	0.565 (*p* < 0.001)
pNN50	–	–	–	–	0.588 (*p* < 0.001)	0.658 (*p* < 0.001)	0.674 (*p* < 0.001)
Pearson correlation coefficient*							
lnSDNN	0.509 (*p* < 0.001)	0.564 (*p* < 0.001)	0.626 (*p* < 0.001)	0.730 (*p* < 0.001)	0.735 (*p* < 0.001)	0.740 (*p* < 0.001)	0.811 (*p* < 0.001)
Cliff’s delta							
SDNN	0.220	0.350	0.435	0.332	0.052	0.279	0.158
RMSSD	0.094	0.200	0.145	0.109	0.068	0.139	0.085
pNN50	–	–	–	–	0.086	0.137	0.092
Cohen’s *d*							
lnSDNN	0.319	0.648	0.723	0.512	0.086	0.460	0.240

**Note:**

* Statistically significant correlation (applied Bonferroni–Holm correction); 10s-1, 10s-2, and 10s-3 denote the first, second, and third group, of 10 s recordings, 30s-1 and 30s-2 the first and second group, of 30 s recordings, 60s the group of 60 s recordings, avg10s the average of all 10 s recordings, and avg30s the average of all 30 s recordings.

## Discussion

In the present study, the agreement between selected UST-PRV metrics (SDNN, lnSDNN, RMSSD, and pNN50) derived from 10 s, 30 s, and 60 s rPPG recordings and the corresponding reference UST-PRV metrics was assessed. The main reason for selecting SDNN, RMSSD, and pNN50 is that previous studies studied the feasibility of assessing PRV using these metrics derived from 10 s, 30 s, or 60 s recordings (Shaffer & Ginsberg, 2017). Moreover, SDNN and RMSSD are two of the most commonly used time-domain HRV/PRV metrics (Malik et al., 1996; Nunan, Sandercock & Brodie, 2010). Most other metrics either require longer recordings and/or are less important for clinical purposes. The reason for selecting the recording lengths of 10 s, 30 s, and 60 s is that 10 s recordings are used in check-ups of patients with atrial fibrillation (Camm et al., 2010), recordings of up to 30 s are being routinely collected, and the duration of the videos in the selected data set was limited to 60 s.

Accurate systolic peak detection and detection of artefacts are crucial for HRV/PRV analysis. When it comes to the filtering of IBIs in rPPG studies, Poh, McDuff & Picard (2011) applied a non-causal variable threshold (NC-VT) (Vila et al., 1997) iterative method. The authors set the tolerance to 30% (Poh, McDuff & Picard, 2011), whereas there is no information on the values of *u*_*n*_ and *u*_*m*_, which depend on the deviation of the heart rate/PR value acquired in the previous iteration step. Therefore, one cannot directly compare the proposed IBIs filtering algorithm with the one applied by Poh, McDuff & Picard (2011). The performance of the proposed algorithm was assessed in terms of the reduction of the MAE of the studied PRV metrics (see Table 3) and by manual inspection of the detected peaks before and after filtering. Based on these results, we were able to justify the application of the proposed IBIs filtering algorithm.

We aimed to evaluate the validity of the rPPG-derived UST-PRV metrics for the raw as well as for the log-transformed data to showcase the effect of log-transformation on the agreement of the results. Log-transformation is one of the most popular methods in biomedical sciences aimed at conforming the data to a normal distribution and reducing its variability, despite its controversy (Feng et al., 2014). The log-transformation itself does namely not guarantee that the transformed data are less variable or follow a normal distribution more closely than the original data (Feng et al., 2014). This fact was confirmed in our study by the result of the Shapiro–Wilk test, which rejected the null hypothesis that lnRMSSD comes from a standard distribution at α = 0.05. Therefore, lnRMSSD data were not included in further analysis. Another controversy of the log-transformation relates to the log-transformed data being strongly dependent on the selected constant that needs to be added to the non-positive values before the actual transformation takes place (Feng et al., 2014). Since the pNN50 data included zero values (in contrast to SDNN and RMSSD), we decided not to apply log-transformation to pNN50. As a result, only lnSDNN was included in further analysis with respect to log-transformed data.

The differences between the SDNN, lnSDNN, RMSSD, and pNN50 values derived from rPPG and the values derived from the corresponding reference (PPG) recordings are most likely due to multiple reasons. First, the pulse transit time (PTT) variability (which indirectly influences PRV) differs between PPG and rPPG due to the different measuring sites of both methods. Second, in PPG, the contact pressure applied to the skin influences the amplitude and shape of the waveform. Third, there might be an effect on PRV due to the different operating modes of both measuring methods used in the data set (transmission mode in PPG and reflectance mode in rPPG). Last but not least, on the rPPG side, the problem relates to the videos being lossless compressed, rather than uncompressed. Most likely, the last one is what is crucial since it influences the raw rPPG signals, which represent the basis of any rPPG research.

In the next part of the study, we assessed to what extent the selected UST-PRV metrics derived from rPPG recordings of different lengths match the UST-PRV metrics derived from the 60 s reference (PPG) recordings. The results given in Tables 6 and 7 and Fig. 3 show that the level of agreement between the rPPG- and PPG-derived SDNN and lnSDNN increases as the recording length increases in terms of correlation, BA plots, and effect size parameters. First, this can be explained by the fact that a single artefact has a more significant influence on the result in shorter recordings compared to longer ones. Second, SDNN reflects all the cyclic components responsible for HRV/PRV, whereas RMSSD and pNN50 only encompass high-frequency components. With shorter recording lengths, the SDNN therefore encompasses shorter cycle lengths and thus depends strongly on the length of the recording (Malik et al., 1996). This finding is in line with the studies examining UST-HRV (Munoz et al., 2015; Nussinovitch et al., 2011). The second reasoning also explains the effect of the stronger agreement with an increasing recording length being more prominent for SDNN/lnSDNN than for RMSSD. When it comes to pNN50 (Tables 6 and 7; Fig. 5), there is no significant change in values of the agreement parameters as the recording length increases. The results given in Tables 6 and 7 also show non-negligible differences between non-overlapping recordings of the same length, which is due to some parts of raw rPPG signals being noisier than others. This fact reflects the innate characteristic of rPPG, that is, the relative amplitude (defined as AC/DC) of the human pulsatile component is very small (e.g. (0.1 × 10^−3^, 2 × 10^−3^)) for the R channel (Verkruysse et al., 2017), making the rPPG signals very susceptible to motion and other artefacts. Therefore, the agreement between the rPPG- and PPG-derived UST-PRV metrics tends to improve by averaging multiple recordings of the same length. A comparison of the results for raw and log-transformed SDNN shows similar agreement in terms of correlation, BA plots, and effect size, but it is hard to compare the parametric measures with the non-parametric ones directly. The limitations of the log-transformation mean that only lnSDNN was evaluated in the present study. To overcome all limitations of the log-transformation, it is suggested that newer analytic methods, such as generalized estimating equations, should be preferred (Feng et al., 2014).

Table 8 presents the results of existing rPPG studies that allow a comparison with the results of this study. In terms of MAE, our results fall into the range of MAE for motion cases provided by Huang & Dung (2016). This observation is somewhat expected since the PURE data set used in the present study contains videos with different scenarios of controlled motion. Huang & Dung (2016), however, relied on small number of recordings (12 recordings of six different subjects), did not provide the distance between the camera (frontal smartphone camera) and the subjects, and used different recordings for the ground truth (ECG). On the other hand, the results of Melchor Rodriguez & Ramos-Castro (2018) outperform our results in terms of correlation and MAE. Still, a key drawback of their study was the application of the bandpass filter that covered a narrower range of the human PR band ((36,120) BPM), which increased the chance of eliminating motion and other artefacts from the studied rPPG signals. The total number of video recordings included in their study is also unclear. Further, none of the aforementioned studies publicly shared their data set, also hindering a comparison of the results. This is one problem that is shared by rPPG studies, which results in different studies using different recording equipment, lighting conditions, recording setups and signal processing algorithms. This problem suggests that at least a partial standardization of the rPPG studies is needed to ensure a fair comparison of the results. It would be also beneficial if the rPPG community were to propose and adopt a common data set that would include facial video recordings in different recording setups together with ECG and PPG reference recordings. This data set could then be used to test and verify of the algorithms for rPPG signal extraction from video recordings.

**Table 8:** Overview of the results of studies assessing SDNN, RMSSD, and pNN50 derived from rPPG signals equal to or less than 60 s. ICC, inter-correlation coefficient; MAE, mean absolute error; ME, mean error; *r*, correlation coefficient.

Study	PRV metric	Result
Huang & Dung (2016)	SDNN	MAE = 0.49*–*3.79 (static); MAE = 2.08*–*28.87 (motion)
	RMMSD	MAE = 0.09–7.97; MAE = 10.29–50.04 (motion)
	SDNN	*r* = 0.9750, ICC = 0.9491, MAE = 3.49 (stationary)
		*r* = 0.9896, ICC = 0.9766, MAE = 2.65 (motion)
Melchor Rodriguez & Ramos-Castro (2018)	RMSSD	*r* = 0.9791, ICC = 0.9208, MAE = 7.13 (stationary)
		*r* = 0.9463, ICC = 0.8898, MAE = 7.81 (motion)
	pNN50	*r* = 0.9350, ICC = 0.8996, MAE = 8.65 (stationary)
		*r* = 0.9204, ICC = 0.8560, MAE = 7.80 (motion)

An important concern about the present study is comparison of rPPG-derived UST-PRV metrics with PPG-derived UST-PRV metrics, rather than with ECG-derived ST-PRV metrics (i.e. the gold standard). Yet we believe that if rPPG is not a valid surrogate for PPG in terms of UST-PRV assessment, then it is hard to expect that rPPG-derived UST-PRV metrics can serve as a valid surrogate for ECG-derived ST-HRV metrics. In contrast to HRV, PRV is namely affected by the variability in pulse transit time, which relates to arterial compliance and blood pressure, and it therefore changes on a beat-to-beat basis (Ma & Zhang, 2006). Since PPG and rPPG signals are not usually measured on the same part of the body (on a finger in PPG and most often on a face in rPPG), PTTs in both methods are different, creating another difference between the two methods, and it is this very difference that we wished to assess in the present study. Second, the variance of HRV/PRV increases with the recording length (Malik et al., 1996). Further, in the case of SDNN, which depends highly on the recording length (Malik et al., 1996), the larger the difference in the length of the two recordings, the less appropriate is the comparison of SDNN values derived from these recordings. Finally, to the best of our knowledge, there is no publicly available appropriate rPPG data set offering at least 5-minute-long reference ECG recordings.

Apart from the selected PURE data set, there are however other data sets that contain uncompressed/lossless compressed videos and could be used instead: the ECG-Fitness database (Špetlík, Franc & Matas, 2018), the MMSE-HR (Tulyakov et al., 2016) and UBFC-RPPG database (Bobbia et al., 2017). Yet the first contains videos of subjects during physical activities, whereas we wanted to initially assess the validity of UST-PRV metrics extracted from videos containing stationary subjects and/or subjects with controlled head motion (to first study the validity of rPPG-derived UST-PRV metrics for subjects in close-to-resting conditions). The second data set requires the payment of handling fees to be given access. The third one, the recently published UBFC-RPPG database (Bobbia et al., 2017) offers fewer videos (50) than the selected data set. We therefore decided to use PURE. For the list of other data sets suitable for general rPPG research, we refer the interested reader to Finžgar & Podržaj (2018). Other limitations of the present study relate to the small size of the studied sample, the short lengths of the video recordings, and the lack of different age groups and skin phototypes included in the data set (all of which relate to the public availability of the rPPG data sets). Lastly, the sampling rate of the reference pulse waveform signals measurement and the camera frame rate of the video recordings from the selected data set did not meet the standard (Malik et al., 1996). However, Choi & Shin (2017) studied the effect of a reduced sampling rate on the reliability of ST-PRV assessment. The authors suggested a sampling rate of 25 Hz as the minimal sampling rate for assessing selected time-domain and frequency-domain PRV metrics (reference data were derived from PPG signals recorded with a sampling rate of 10 kHz). Recently, Fujita & Suzuki (2019) assessed the effect of a lower sampling rate on the estimation of PPG waveform features. The study shows that height of the systolic peak, which is relevant to our study since this peak was used for the extraction of IBIs and consequent PRV analysis, is stable even at a sampling rate of 10 Hz. The authors acquired signals of lower sampling rates by downsampling the PPG signals recorded at 240 Hz (reference signals). Based on the findings of these two studies, we were able to justify the selection of PURE data set for the analysis of rPPG-derived UST-PRV metrics.

In order to overcome the limitations related to the data set used, the creation of a publicly available data set with the following characteristics should be considered in future studies: a minimum of 30 studied subjects of various skin phototypes and ages, at least 5-minute-long reference ECG recordings, various recording setups (motion, varying illuminance, etc.), and availability of uncompressed videos. One expects that such a data set would influence the results of the present study. First, uncompressed videos could increase SNR due to information not being lost during the video compression process. Second, different motion scenarios and varying levels of illuminance would strongly affect SNR as well as the performance of the frontal face detector, which is a crucial first step in the extraction of raw RGB signals from videos. This could be overcome by applying a different approach to the detection of skin pixels of interest, such as using a living-skin detector (Wang, Stuijk & De Haan, 2017). When lighting conditions are poor, a near-infrared camera system (Van Gastel, Stuijk & De Haan, 2015) could be used to overcome these conditions. Similarly, the presence of subjects of different skin phototypes and ages would affect SNR (De Haan & Jeanne, 2013; Moço, Stuijk & De Haan, 2016) due to the different extent of skin inhomogeneity and melanin concentrations. Finally, a bigger number of subjects would increase the inference validity. To further justify the use of rPPG in UST-PRV analysis (1) the agreement between the UST-PRV metrics and ST-PRV metrics based on 5-minute-long recordings; and (2) the agreement between ECG-derived UST-HRV and rPPG-derived (U)ST-PRV needs to be thoroughly assessed in future research.

## Conclusions

Ultra-short-term-PRV metrics derived from rPPG recordings (extracted from lossless compressed videos) of 10 s, 30 s, and 60 s in length manage to capture UST-PRV metrics derived from reference (PPG) recordings well. The agreement, which is the highest for SDNN (followed by pNN50 and RMMSD) increases as the recording length increases. This finding was subjected to thorough statistical analysis. The study also showcased the problems related to the log-transformation of data and lack of standardization of rPPG studies, which can hinder the objective comparison of the results coming from different studies. Despite this, it is still anticipated that UST-PRV analysis from video recordings will in the future play an important role in various healthcare applications.

## Supplemental Information

10.7717/peerj.8342/supp-1 Supplemental Information 1 Results of multiple pairwise comparison of the group means using Bonferroni critical values for one-way ANOVA.

10.7717/peerj.8342/supp-2 Supplemental Information 2 MATLAB scripts used for the production of the results presented in the manuscript. Please open instructions.txt enclosed in the .zip file.

## Additional Information and Declarations

### Data Availability

The following information was supplied regarding data availability: All MATLAB scripts used in the production of the results are available as Supplemental Files.
