# Supplementary material for: Feasibility of assessing ultra-short-term pulse rate variability from video recordings
Source: PeerJ. 2020 Jan 7;8:e8342. doi: 10.7717/peerj.8342 (PMC6953345; doi:10.7717/peerj.8342)
Supplement: Supplemental Information 1 [file peerj-08-8342-s001.docx]

| **SDNN** | | | | | |
| --- | --- | --- | --- | --- | --- |
| **Group 1 index** | **Group 2 index** | **mean difference** | **lower 95% limit** | **upper 95% limit** | **p-value** |
| 60s PPG | 10s-1 rPPG | -8.6 | 3.8 | 16.3 | 1 |
| 60s PPG | 10s-2 rPPG | -2.6 | 9.9 | 22.3 | 0.3922 |
| 60s PPG | 10s-3 rPPG | -1.7 | 10.8 | 23.2 | 0.2045 |
| 60s PPG | avg10s rPPG | -4.3 | 8.2 | 20.6 | 1 |
| 60s PPG | 30s-1 rPPG | -11.9 | 0.6 | 13.0 | 1 |
| 60s PPG | 30s-2 rPPG | -5.3 | 7.1 | 19.6 | 1 |
| 60s PPG | avg30s rPPG | -8.6 | 3.9 | 16.3 | 1 |
| 60s PPG | 60s rPPG | -12.3 | 0.1 | 12.6 | 1 |
| 10s-1 rPPG | 10s-2 rPPG | -6.4 | 6.1 | 18.5 | 1 |
| 10s-1 rPPG | 10s-3 rPPG | -5.5 | 6.9 | 19.4 | 1 |
| 10s-1 rPPG | avg10s rPPG | -8.1 | 4.3 | 16.8 | 1 |
| 10s-1 rPPG | 30s-1 rPPG | -15.7 | -3.2 | 9.2 | 1 |
| 10s-1 rPPG | 30s-2 rPPG | -9.1 | 3.3 | 15.7 | 1 |
| 10s-1 rPPG | avg30s rPPG | -12.4 | 0.0 | 12.5 | 1 |
| 10s-1 rPPG | 60s rPPG | -16.1 | -3.7 | 8.7 | 1 |
| 10s-2 rPPG | 10s-3 rPPG | -11.6 | 0.9 | 13.3 | 1 |
| 10s-2 rPPG | avg10s rPPG | -14.2 | -1.7 | 10.7 | 1 |
| 10s-2 rPPG | 30s-1 rPPG | -21.8 | -9.3 | 3.1 | 0.5957 |
| 10s-2 rPPG | 30s-2 rPPG | -15.2 | -2.8 | 9.7 | 1 |
| 10s-2 rPPG | avg30s rPPG | -18.5 | -6.0 | 6.4 | 1 |
| 10s-2 rPPG | 60s rPPG | -22.2 | -9.8 | 2.7 | 0.4271 |
| 10s-3 rPPG | avg10s rPPG | -15.0 | -2.6 | 9.8 | 1 |
| 10s-3 rPPG | 30s-1 rPPG | -22.6 | -10.2 | 2.3 | 0.3199 |
| 10s-3 rPPG | 30s-2 rPPG | -16.1 | -3.6 | 8.8 | 1 |
| 10s-3 rPPG | avg30s rPPG | -19.3 | -6.9 | 5.5 | 1 |
| 10s-3 rPPG | 60s rPPG | -23.1 | -10.6 | 1.8 | 0.224 |
| avg10s rPPG | 30s-1 rPPG | -20.0 | -7.6 | 4.9 | 1 |
| avg10s rPPG | 30s-2 rPPG | -13.5 | -1.0 | 11.4 | 1 |
| avg10s rPPG | avg30s rPPG | -16.8 | -4.3 | 8.1 | 1 |
| avg10s rPPG | 60s rPPG | -20.5 | -8.0 | 4.4 | 1 |
| 30s-1 rPPG | 30s-2 rPPG | -5.9 | 6.5 | 19.0 | 1 |
| 30s-1 rPPG | avg30s rPPG | -9.2 | 3.3 | 15.7 | 1 |
| 30s-1 rPPG | 60s rPPG | -12.9 | -0.5 | 12.0 | 1 |
| 30s-2 rPPG | avg30s rPPG | -15.7 | -3.3 | 9.2 | 1 |
| 30s-2 rPPG | 60s rPPG | -19.4 | -7.0 | 5.4 | 1 |
| avg30s rPPG | 60s rPPG | -16.2 | -3.7 | 8.7 | 1 |

| **lnSDNN** | | | | | |
| --- | --- | --- | --- | --- | --- |
| **Group 1 index** | **Group 2 index** | **mean difference** | **lower 95% limit** | **upper 95% limit** | **p-value** |
| 60s PPG | 10s-1 rPPG | -0.1303 | 0.1421 | 0.4145 | 1 |
| 60s PPG | 10s-2 rPPG | 0.0203 | 0.2927 | 0.5651 | 0.02151 |
| 60s PPG | 10s-3 rPPG | 0.0593 | 0.3317 | 0.6041 | 0.00371 |
| 60s PPG | avg10s rPPG | -0.0714 | 0.2010 | 0.4734 | 0.6495 |
| 60s PPG | 30s-1 rPPG | -0.2374 | 0.0350 | 0.3074 | 1 |
| 60s PPG | 30s-2 rPPG | -0.0839 | 0.1885 | 0.4609 | 0.95606 |
| 60s PPG | avg30s rPPG | -0.1794 | 0.0930 | 0.3654 | 1 |
| 60s PPG | 60s rPPG | -0.2546 | 0.0178 | 0.2902 | 1 |
| 10s-1 rPPG | 10s-2 rPPG | -0.1217 | 0.1507 | 0.4231 | 1 |
| 10s-1 rPPG | 10s-3 rPPG | -0.0828 | 0.1896 | 0.4620 | 0.92299 |
| 10s-1 rPPG | avg10s rPPG | -0.2134 | 0.0590 | 0.3314 | 1 |
| 10s-1 rPPG | 30s-1 rPPG | -0.3794 | -0.1070 | 0.1654 | 1 |
| 10s-1 rPPG | 30s-2 rPPG | -0.2260 | 0.0464 | 0.3188 | 1 |
| 10s-1 rPPG | avg30s rPPG | -0.3214 | -0.0490 | 0.2234 | 1 |
| 10s-1 rPPG | 60s rPPG | -0.3966 | -0.1242 | 0.1482 | 1 |
| 10s-2 rPPG | 10s-3 rPPG | -0.2334 | 0.0390 | 0.3114 | 1 |
| 10s-2 rPPG | avg10s rPPG | -0.3641 | -0.0917 | 0.1807 | 1 |
| 10s-2 rPPG | 30s-1 rPPG | -0.5301 | -0.2577 | 0.0147 | 0.08919 |
| 10s-2 rPPG | 30s-2 rPPG | -0.3766 | -0.1042 | 0.1681 | 1 |
| 10s-2 rPPG | avg30s rPPG | -0.4721 | -0.1997 | 0.0727 | 0.67751 |
| 10s-2 rPPG | 60s rPPG | -0.5473 | -0.2749 | -0.0025 | 0.04517 |
| 10s-3 rPPG | avg10s rPPG | -0.4031 | -0.1307 | 0.1417 | 1 |
| 10s-3 rPPG | 30s-1 rPPG | -0.5691 | -0.2967 | -0.0243 | 0.01815 |
| 10s-3 rPPG | 30s-2 rPPG | -0.4156 | -0.1432 | 0.1292 | 1 |
| 10s-3 rPPG | avg30s rPPG | -0.5110 | -0.2387 | 0.0337 | 0.18153 |
| 10s-3 rPPG | 60s rPPG | -0.5863 | -0.3139 | -0.0415 | 0.00847 |
| avg10s rPPG | 30s-1 rPPG | -0.4384 | -0.1660 | 0.1064 | 1 |
| avg10s rPPG | 30s-2 rPPG | -0.2849 | -0.0125 | 0.2599 | 1 |
| avg10s rPPG | avg30s rPPG | -0.3804 | -0.1080 | 0.1644 | 1 |
| avg10s rPPG | 60s rPPG | -0.4556 | -0.1832 | 0.0892 | 1 |
| 30s-1 rPPG | 30s-2 rPPG | -0.1189 | 0.1535 | 0.4258 | 1 |
| 30s-1 rPPG | avg30s rPPG | -0.2144 | 0.0580 | 0.3304 | 1 |
| 30s-1 rPPG | 60s rPPG | -0.2896 | -0.0172 | 0.2552 | 1 |
| 30s-2 rPPG | avg30s rPPG | -0.3678 | -0.0954 | 0.1770 | 1 |
| 30s-2 rPPG | 60s rPPG | -0.4431 | -0.1707 | 0.1017 | 1 |
| avg30s rPPG | 60s rPPG | -0.3476 | -0.0752 | 0.1972 | 1 |

| **RMSSD** | | | | | |
| --- | --- | --- | --- | --- | --- |
| **Group 1 index** | **Group 2 index** | **mean difference** | **lower 95% limit** | **upper 95% limit** | **p-value** |
| 60s PPG | 10s-1 rPPG | -14.6 | 1.2 | 17.1 | 1 |
| 60s PPG | 10s-2 rPPG | -10.9 | 5.0 | 20.8 | 1 |
| 60s PPG | 10s-3 rPPG | -9.5 | 6.4 | 22.2 | 1 |
| 60s PPG | avg10s rPPG | -11.6 | 4.2 | 20.0 | 1 |
| 60s PPG | 30s-1 rPPG | -15.6 | 0.2 | 16.1 | 1 |
| 60s PPG | 30s-2 rPPG | -12.4 | 3.4 | 19.3 | 1 |
| 60s PPG | avg30s rPPG | -14.0 | 1.8 | 17.7 | 1 |
| 60s PPG | 60s rPPG | -16.0 | -0.1 | 15.7 | 1 |
| 10s-1 rPPG | 10s-2 rPPG | -12.1 | 3.7 | 19.5 | 1 |
| 10s-1 rPPG | 10s-3 rPPG | -10.7 | 5.1 | 20.9 | 1 |
| 10s-1 rPPG | avg10s rPPG | -12.9 | 2.9 | 18.8 | 1 |
| 10s-1 rPPG | 30s-1 rPPG | -16.8 | -1.0 | 14.8 | 1 |
| 10s-1 rPPG | 30s-2 rPPG | -13.6 | 2.2 | 18.0 | 1 |
| 10s-1 rPPG | avg30s rPPG | -15.2 | 0.6 | 16.4 | 1 |
| 10s-1 rPPG | 60s rPPG | -17.2 | -1.4 | 14.4 | 1 |
| 10s-2 rPPG | 10s-3 rPPG | -14.4 | 1.4 | 17.2 | 1 |
| 10s-2 rPPG | avg10s rPPG | -16.6 | -0.8 | 15.0 | 1 |
| 10s-2 rPPG | 30s-1 rPPG | -20.5 | -4.7 | 11.1 | 1 |
| 10s-2 rPPG | 30s-2 rPPG | -17.4 | -1.5 | 14.3 | 1 |
| 10s-2 rPPG | avg30s rPPG | -18.9 | -3.1 | 12.7 | 1 |
| 10s-2 rPPG | 60s rPPG | -20.9 | -5.1 | 10.7 | 1 |
| 10s-3 rPPG | avg10s rPPG | -18.0 | -2.2 | 13.7 | 1 |
| 10s-3 rPPG | 30s-1 rPPG | -21.9 | -6.1 | 9.7 | 1 |
| 10s-3 rPPG | 30s-2 rPPG | -18.7 | -2.9 | 12.9 | 1 |
| 10s-3 rPPG | avg30s rPPG | -20.3 | -4.5 | 11.3 | 1 |
| 10s-3 rPPG | 60s rPPG | -22.3 | -6.5 | 9.3 | 1 |
| avg10s rPPG | 30s-1 rPPG | -19.8 | -3.9 | 11.9 | 1 |
| avg10s rPPG | 30s-2 rPPG | -16.6 | -0.8 | 15.1 | 1 |
| avg10s rPPG | avg30s rPPG | -18.2 | -2.4 | 13.5 | 1 |
| avg10s rPPG | 60s rPPG | -20.1 | -4.3 | 11.5 | 1 |
| 30s-1 rPPG | 30s-2 rPPG | -12.6 | 3.2 | 19.0 | 1 |
| 30s-1 rPPG | avg30s rPPG | -14.2 | 1.6 | 17.4 | 1 |
| 30s-1 rPPG | 60s rPPG | -16.2 | -0.4 | 15.5 | 1 |
| 30s-2 rPPG | avg30s rPPG | -17.4 | -1.6 | 14.2 | 1 |
| 30s-2 rPPG | 60s rPPG | -19.4 | -3.6 | 12.3 | 1 |
| avg30s rPPG | 60s rPPG | -17.8 | -2.0 | 13.9 | 1 |

| **pNN50** | | | | | |
| --- | --- | --- | --- | --- | --- |
| **Group 1 index** | **Group 2 index** | **mean difference** | **lower 95% limit** | **upper 95% limit** | **p-value** |
| 60s PPG | 30s-1 rPPG | -6.0 | 3.3 | 12.5 | 1 |
| 60s PPG | 30s-2 rPPG | -5.6 | 3.7 | 12.9 | 1 |
| 60s PPG | avg30s rPPG | -5.8 | 3.5 | 12.7 | 1 |
| 60s PPG | 60s rPPG | -5.9 | 3.4 | 12.6 | 1 |
| 30s-1 rPPG | 30s-2 rPPG | -8.8 | 0.4 | 9.7 | 1 |
| 30s-1 rPPG | avg30s rPPG | -9.1 | 0.2 | 9.5 | 1 |
| 30s-1 rPPG | 60s rPPG | -9.1 | 0.1 | 9.4 | 1 |
| 30s-2 rPPG | avg30s rPPG | -9.5 | -0.2 | 9.1 | 1 |
| 30s-2 rPPG | 60s rPPG | -9.6 | -0.3 | 9.0 | 1 |
| avg30s rPPG | 60s rPPG | -9.4 | -0.1 | 9.2 | 1 |
